# A Fee-Splitting Law

**Published:** 1915-02

**Authors:** 


### A Fee-Splitting Law.

Some years ago the United States government made it an offense for physicians practicing within the Hot Springs reservation, in Arkansas, either to pay or receive commissions for professional services. There the evil had grown to such proportions that drummers were sent out on all the trains approaching the famous resort, and these drummers, the most skillful of their kind, were very sure to land, on each and every trip, a victim in the hands of their employers.

This action has entirely eradicated this vicious practice, with the result that Hot Springs has taken its position as a highly respectable and thoroughly ethical health resort, to which people may go for treatment with the assurance that they will come in contact with an honorable profession, the status and quality of which has been assured by the wise attitude of the national government.

This precedent has demonstrated the possibility of dealing with this evil elsewhere. The fact that the practice has grown to a very considerable extent between certain physicians and certain consultants has been the one conspicuous scandal of the medical profession in the last decade. The people, however, are beginning to understand that, in going into the offices of one of these certain physicians, they are liable to be sold as so many chattels into the hands of the alleged consultant, whose leading qualification is the fact that he will pay to the original attendant or purveyor the largest possible commission. The moment that this practice became established, that moment the high integrity of those addicted to it became a thing of the past. Fortunately, the time never was and never will be when such venality can, with justice, be recognized as the ethical expression of the medical profession. Those members of the profession who have been bold enough to speak out on this subject, to denounce the fee-splitter, to denounce fee-splitting and to denounce the despicable smallness of everybody connected with such transactions, have succeeded in arousing the only possible remedial movement. The very moment that the people themselves found that certain doctors were addicted to this secret practice, and that they, as patients, were not safe in the hands of such unsuspected individuals, that moment they began a movement looking to their own protection. This movement has crystallized in bills now before practically every Legislature of the United States. In two States, at least, namely, Wisconsin and Nebraska, these bills have been enacted into laws.

The laws, as framed and passed, seem to get at the very heart of the question. They apply to any physician or surgeon who shall claim, or demand, or collect, or receive any money which can be construed as a commission. They apply alike to the medical attendant and to the consultant, whether the consultant is a medical man or a surgeon. The offense is made a fraud punishable by a fine or by imprisonment. The most serious phase of the penal clause is that which provides that offenders under this law shall be subject to revocation of license to practice. In the Wisconsin law there is a paragraph which makes the measure applicable to offenders living in neighboring or distant States.

Now that the people have taken this matter into their own hands, it is of the highest importance that respectable members of the medical profession shall rally to their support. No man who has the welfare of the people, the honor and integrity of his profession, or his own self-respect at heart, can fail to give his strongest possible aid to so beneficent a measure. The matter should be brought to the attention of all the people through the avenues not only of the medical press, but of every newspaper of general or local circulation, all of which should be asked to contribute to the desirable end in view. Not only the press, but the pulpit and the schools and every other instrumentality by which public opinion is formed and legislative action is determined should be invoked to lend a helping hand not only until the law is enacted but until every offender under its provisions shall be run out of the profession.—*Lancet-Clinic.*

